# Quantitative Analysis of the Interdisciplinarity of Applied Mathematics

**DOI:** 10.1371/journal.pone.0137424

**Published:** 2015-09-09

**Authors:** Zheng Xie, Xiaojun Duan, Zhenzheng Ouyang, Pengyuan Zhang

**Affiliations:** College of Science, National University of Defense Technology, Changsha, Hunan, China; Max Planck Institute for the Physics of Complex Systems, GERMANY

## Abstract

The increasing use of mathematical techniques in scientific research leads to the interdisciplinarity of applied mathematics. This viewpoint is validated quantitatively here by statistical and network analysis on the corpus PNAS 1999–2013. A network describing the interdisciplinary relationships between disciplines in a panoramic view is built based on the corpus. Specific network indicators show the hub role of applied mathematics in interdisciplinary research. The statistical analysis on the corpus content finds that algorithms, a primary topic of applied mathematics, positively correlates, increasingly co-occurs, and has an equilibrium relationship in the long-run with certain typical research paradigms and methodologies. The finding can be understood as an intrinsic cause of the interdisciplinarity of applied mathematics.

## Introduction

Interdisciplinary research means that data, techniques, concepts, and theories from two or more disciplines are integrated to solve problems whose solutions are beyond the scope of a single discipline or area of research practice [1, 2]. Mathematical science plays an important role in interdisciplinary research, because many problems in various disciplines of physical science, biological science, and social science are using increasingly mathematical techniques [3]. The increasing application of mathematical theories and methods to other disciplines have therefore led to the development of mathematical science, especially applied mathematics [4].

The panoramic view of the relationships between disciplines can be drawn as a network, regarding the disciplines as nodes and the interdisciplinary relationships as edges. The network is built here by the disciplinary information of the papers published in the Proceedings of the National Academy of Sciences (PNAS, http://www.pnas.org) in 1999–2013. Two disciplines are connected if there is a paper belonging to them both. Then, the interdisciplinarity of disciplines is quantitatively expressed by the network indicators about the strength and breadth of the connections between disciplines, such as degree, betweenness centrality [5], etc. Those indicators show that applied mathematics not only widely and directly participates in interdisciplinary research, but also makes bridges for carrying interdisciplinary research between other disciplines.

In order to get a more comprehensive understanding of the interdisciplinarity of applied mathematics, we analyze the contents of the papers. The tests of cointegration and correlation on the quarterly numbers of papers containing certain topic words, e.g. “algorithm”, show that the development of algorithms and that of certain research paradigms [6–9] (model, experiment, simulation, and data-driven) and transdisciplinary topics [10–12] (system, network, and control) obey equilibrium relationships in the long-run, and are positively correlated. The co-word occurrence analysis shows the increasing trends of algorithmization of those research paradigms and transdisciplinary topics. Those found relationships can be considered as causes of the interdisciplinarity of applied mathematics.

This paper is organized as follows. The data processing is introduced in Section 2. The network analysis is shown in Section 3. The statistical analysis is presented in Section 4. The conclusion is drawn in Section 5.

## Data processing

**Fig 1:**
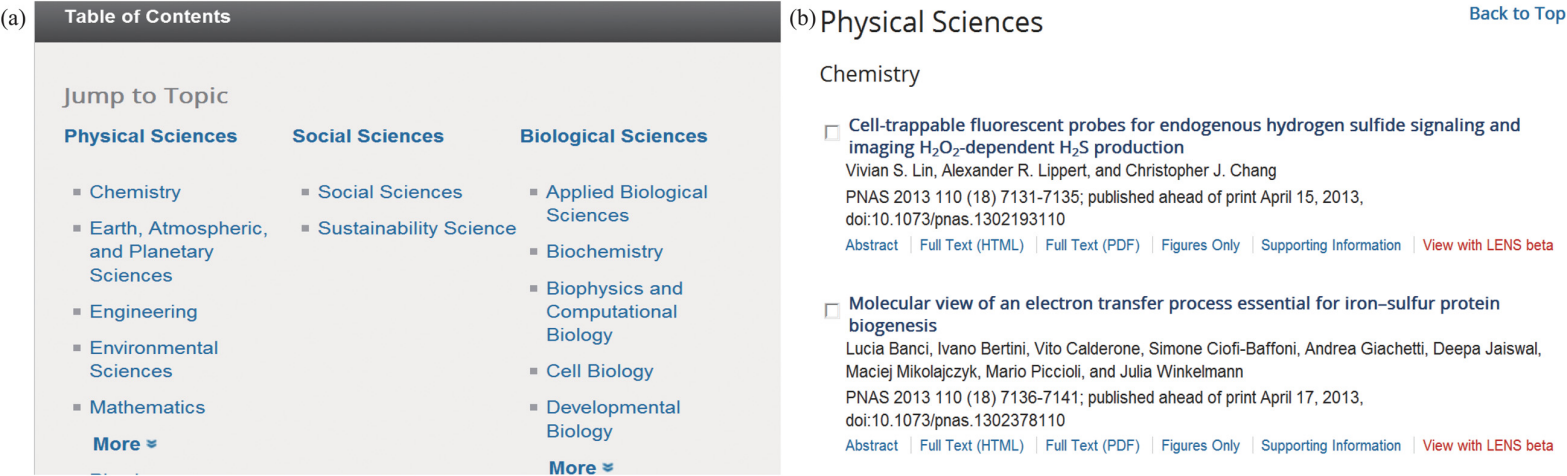
The discipline information given by PNAS. The panels (a,b) respectively come from http://www.pnas.org/content/110/18.toc, http://www.pnas.org/content/110/18.toc#PhysicalSciences.

The journal PNAS publishes high quality research reports, commentaries, reviews, perspectives and letters. The corpus analyzed here consists of 52,803 papers published in PNAS in 1999–2013. The journal provided the discipline information of the papers (Fig 1). There are 3 first level disciplines, viz. biological science, physical science, and social science, and 39 second level disciplines, such as mathematics, computer science, etc. So the papers can be classified according to their discipline information.

Most of the papers have been classified by the first and second level disciplines. Some papers are only classified by the first level disciplines. For those papers, we considered their second level discipline to be the same as their first level one. Hence we added the first level disciplines into the set of second level disciplines. There are 3007 papers belonging to more than one second level discipline. For example, Ref [13] belongs to applied mathematics and ecology. Those papers can be considered to be interdisciplinary papers. The discipline information of the papers will be used to build a network describing the interdisciplinary relationships between disciplines in Section 3.

Many papers have used mathematical techniques, but are not classified into applied mathematics. Thus, we should analyze the contents of the papers. The python package Natural Language Toolkit (NLTK, http://www.nltk.org) is used to build the dictionary for the corpus by its function of morphological reduction. The dictionary contains 31,542 words (S1 Text). Those words belong to the lexicon of NLTK, which includes the English WordNet. Based on the dictionary, the document-term matrix for the corpus is generated, in which the rows correspond to the papers in the corpus and columns correspond to the words. Together with the publication dates of the papers, the quarterly numbers of the papers containing certain words are extracted for analyzing the relationships of algorithms to certain research paradigms and transdisciplinary topics in Section 4.

## Network analysis of the interdisciplinarity of applied mathematics

**Fig 2:**
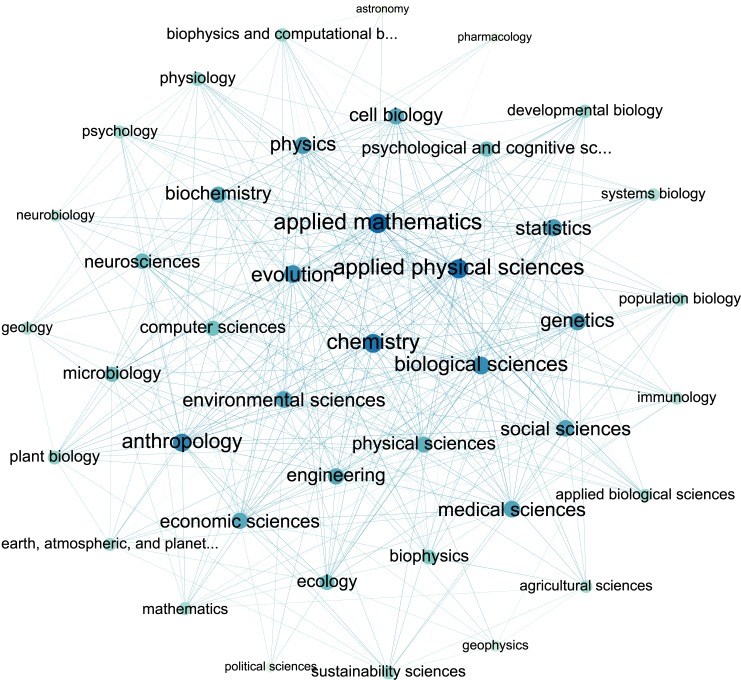
The discipline network. It contains 42 nodes and 354 edges. Two disciplines are connected if there is a paper in PNAS 1999-2013 belonging to them simultaneously.

Based on the discipline information of the corpus, a network describing the connections among disciplines is constructed (The discipline network, Fig 2), in which the nodes are the second level disciplines, and two disciplines are connected if there is a paper belonging to them both. For example, applied mathematics and ecology are connected, because Ref [13] belongs to them both. The network is connected, which means no discipline is isolated. The edges of the network can be assigned weights: the number of interdisciplinary papers between two connected disciplines. The network data is provided in S1 Network.

The phenomenon of the dense relationships between disciplines is quantitatively described by the network indicators [5], viz. the average clustering coefficient 0.55, the diameter 3, the average (weighted) degree 16.87 (148.38), and the graph density 0.41. Those indicators also show the small-world property of the discipline network.

The interdisciplinary breadth and centrality of a discipline can be quantitatively described by the degree and betweenness centrality of the corresponding node in the unweighted discipline network respectively. The degree of a node is the number of nodes connecting to it. The betweenness centrality relates to the number of shortest paths from all nodes to all others that pass through that node. If item transfer through the network follows the shortest paths, a node with high betweenness centrality has a large influence on the transfer behavior.

The interdisciplinary strength of a discipline can be expressed by the number of the interdisciplinary papers involving with that discipline, namely the degree of the corresponding node in the weighted discipline network. PageRank also gives a rough estimate of the importance of nodes (receive more connections from other nodes) in a given network. Hence the interdisciplinary breadth and strength of a discipline can be expressed by the PageRank value of the corresponding node in the unweighted and weighted discipline network respectively.

**Fig 3:**
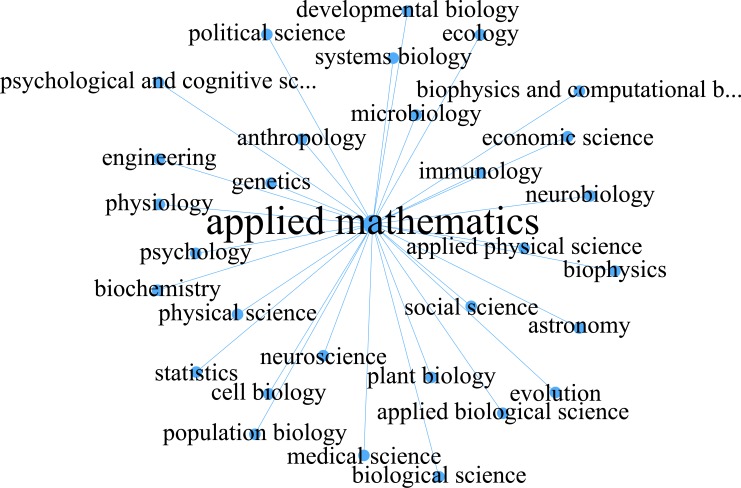
The neighbors of applied mathematics in the discipline network. A discipline connects to applied mathematics if there is a paper in PNAS 1999-2013 belonging to that discipline and applied mathematics simultaneously.

The degree, PageRank and betweenness centrality of applied mathematics in the unweighted network are the highest (Table 1). The degree of applied mathematics is 30, which means the theories and methods of applied mathematics have been directly used by 73.17% of the second level disciplines listed by PNAS, and members of all 3 first level disciplines (Fig 3). The highest value of betweenness centrality means that applied mathematics is a hub node for transferring the ideas, theories, and methods from one discipline to others, and then making bridges for carrying on interdisciplinary research between other disciplines. For example, network cosmology and its application [14–17] are typical interdisciplinary works among the theory of relativity, network science, and scientometrics, which are connected by geometry.

**Table 1:** Certain quantitative indicators for the interdisciplinarity of disciplines.

Disciplinary	*K*	*M*	*N*	*C*	*B*	*S*	*P*	*K*_*W*_	*P*_*W*_
Applied mathematics	**30**	191	380	**15.08**	**53.42**	0.50	**0.04**	195	0.03
Statistics	23	90	146	14.18	21.69	0.62	0.03	92	0.02
Computer science	18	77	101	13.72	9.42	**0.76**	0.03	78	0.01
Engineering	21	217	392	11.62	14.08	0.55	0.03	225	0.03
Economic science	21	94	171	11.54	18.68	0.55	0.03	94	0.02
Applied physical science	29	309	816	10.98	28.80	0.38	**0.04**	314	0.04
Social science	22	78	167	10.28	13.30	0.47	0.03	89	0.02
Psychological and cognitive science	18	160	487	5.91	5.56	0.33	0.02	164	0.03
Environmental science	22	184	695	5.82	22.85	0.26	0.03	186	0.03
Anthropology	26	114	556	5.33	40.71	0.21	**0.04**	116	0.02
Geology	11	137	285	5.29	2.60	0.48	0.02	137	0.02
Sustainability science	13	123	399	4.01	8.40	0.31	0.02	129	0.03
Biophysics and computational biology	13	468	1532	3.97	9.18	0.31	0.02	481	0.06
Earth, atmospheric, and planetary sciences	12	78	243	3.85	2.02	0.32	0.02	82	0.02
Chemistry	28	**1003**	**8645**	3.25	49.61	0.12	**0.04**	**1015**	**0.13**
Ecology	18	162	1084	2.69	11.03	0.15	0.03	167	0.03
Evolution	25	233	2274	2.56	28.53	0.10	0.03	235	0.04
Systems biology	11	36	159	2.49	1.64	0.23	0.02	36	0.01
Psychology	12	83	449	2.22	3.43	0.18	0.02	83	0.02
Applied biological science	12	135	767	2.11	2.51	0.18	0.02	137	0.02
Political science	5	7	17	2.06	0.39	0.41	0.01	7	0
Biological science	25	66	840	1.96	20.24	0.08	0.03	104	0.02
Population biology	12	27	166	1.95	4.18	0.16	0.02	27	0.01
Biophysics	16	359	3957	1.45	6.80	0.09	0.02	359	0.05
Neuroscience	19	290	4398	1.25	14.59	0.07	0.03	296	0.05
Biochemistry	21	333	6303	1.11	17.01	0.05	0.03	335	0.04
Physics	23	229	4818	1.09	18.12	0.05	0.03	229	0.03
Agricultural science	11	22	226	1.07	4.02	0.10	0.02	23	0.01
Geophysics	7	23	175	0.92	1.32	0.13	0.01	23	0.01
Genetics	23	103	2664	0.89	14.67	0.04	0.03	105	0.02
Medical science	22	181	4784	0.83	12.90	0.04	0.03	181	0.03
Cell biology	21	135	3717	0.76	15.50	0.04	0.03	139	0.02
Microbiology	18	92	2812	0.59	11.31	0.03	0.03	92	0.02
Physical science	20	21	835	0.50	7.87	0.03	0.03	56	0.01
Physiology	14	33	1116	0.41	6.07	0.03	0.02	33	0.01
Mathematics	12	18	561	0.39	3.29	0.03	0.02	18	0.01
Developmental biology	13	33	1525	0.28	1.82	0.02	0.02	33	0.01
Plant biology	14	27	1700	0.22	4.74	0.02	0.02	29	0.01
Astronomy	3	3	50	0.18	0.11	0.06	0.01	3	0
Pharmacology	4	26	594	0.18	0.07	0.04	0.01	26	0.01
Immunology	11	43	3070	0.15	1.69	0.01	0.02	43	0.01
Neurobiology	9	16	1003	0.14	0.84	0.02	0.01	16	0.01

The degree, PageRank and betweenness centrality of the nodes in the unweighted (weighted) discipline network are denoted by *K* (*K*_*W*_), *P* (*P*_*W*_), and *B* respectively. The interdisciplinary strength is *S* = *M*/*N* and the cross indicator is *C* = *SK*, where *N* is the number of the papers and *M* is the number of the interdisciplinary papers of a certain discipline in PNAS 1999–2013.

The degree and PageRank of the discipline of chemistry in the weighted network are the highest, which means the interdisciplinary strength of chemistry is the highest. Those indicators of applied mathematics are low, comparing with those of chemistry. This is caused by that PNAS only published a few applied mathematical papers (350 papers in 1999–2013), comparing with the papers of chemistry (8,645 papers in 1999–2013). So we need a more fair indicator to measure the interdisciplinary strength, which is defined as follows.

The relative interdisciplinary strength *S*(*i*) of discipline *i* is defined here as *S*(*i*) = *M*(*i*)/*N*(*i*), where *N*(*i*) is the number of papers of discipline *i* in the corpus, and *M*(*i*) is the number of interdisciplinary papers in discipline *i*. A simple proxy considering both the interdisciplinary strength and breadth is *C*(*i*) = *S*(*i*)*K*(*i*), where *K*(*i*) is the degree of *i* in the discipline network. The proxy is named the cross indicator. Notice that, for certain discipline *i*, e.g. applied mathematics, *M*(*i*) is slight less than the weighted degree *K*_*W*_(*i*) (Table 1). This is caused by that some papers belong to more than two disciplines.

Sort the disciplines by the cross indicator (Table 1). The top three are applied mathematics, statistics in mathematical science, and computer science (whose theory closely relates to mathematical science). The reasons for the high cross indicators differ in different disciplines. Applied mathematics, statistics, computer science, and applied physical science are “output type” disciplines. The ideas and theories of those disciplines have provided a growing arsenal of methods for all of the sciences. Engineering, social science, and economic science are “input type” disciplines. Those disciplines integrate data, techniques, theories, etc. from other disciplines to create new approaches for their problems whose solutions are beyond their own scope.

The high values of the aforementioned indicators in applied mathematics are due to the increasing use of mathematical techniques in scientific research. A growing body of work in physics or computer science is indistinguishable from research done by mathematicians, and similar overlap occurs with medical science, astronomy, economic sciences, and an increasing number of fields. It is difficult today to find any discipline that does not have connections to mathematics, even political science [18].

## Statistical analysis of the relationships of typical research paradigms and methodologies to algorithms

**Fig 4:**
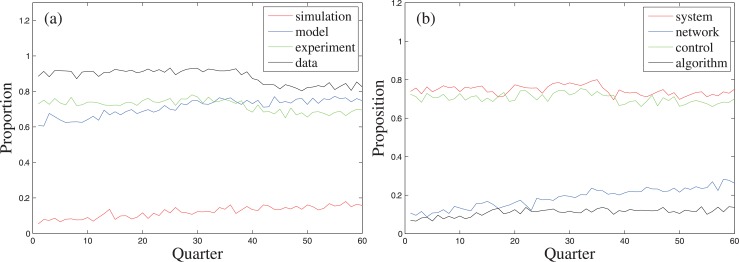
The quarterly proportions of the papers containing a certain topic word. The topic words respectively represent four research paradigms, viz. model, experiment, simulation, and data-driven, and three transdisciplinary topics, viz. system, network, and control.

To understand the underlying causes of the interdisciplinarity of applied mathematics, we discuss the relationships of some typical research paradigms and methodologies to applied mathematics by statistically analyzing the corpus content. A paper containing a topic word means the topic expressed by the word is used or discussed by that paper [19]. The topic words expressing the four basic research paradigms (model, experiment, simulation, and data driven) and the methodologies given by the three typical transdisciplinary topics (system, network and control) can be considered to be “model”, “experiment”, “simulation”, “data”, “system”, “network”, and “control” respectively. For each topic word, the high or increasing proportion of the papers containing that word at certain levels reflects the typicality of the corresponding research paradigm or transdisciplinary topic (Fig 4).

There are 31,542 words appearing in the corpus and also belonging to the lexicon of NLTK, in which there are 976 words appearing in more than 10% of papers (S1 Text). We manually selected typical topic words of applied mathematics from the 976 words, and found the word “algorithm”, which appears in 11.34% of papers. The relationship of a research paradigm or a transdisciplinary topic to algorithms, at certain degrees, can be expressed by the cointegration and correlation between the quarterly numbers of the papers containing the corresponding word and that of the papers containing “algorithm” (S1 Table).

Let the scalars of nominal significance levels of the following tests be 0.05. The augmented Dickey-Fuller test [20] (maxlags = 3) shows that all of the time series in S1 Table are first order integrated. The Johansen test [21] shows that almost all of the time series pairs in Table 2 are cointegrated. This means that, based on the 60 quarters of data from PNAS 1999-2013, the development of algorithms and that of any one of the mentioned research paradigms or transdisciplinary topics obey an equilibrium relationship in the long-run in the academic system.

**Table 2:** The boolean decisions of the Johansen test on certain time series pairs.

	system	network	control	model	experiment	simulation	data
network	1						
control	1	1					
model	1	1	1				
experiment	0	1	1	1			
simulation	1	1	1	1	1		
data	0	1	1	1	1	1	
algorithm	1	1	1	1	1	1	1

When doing the test, we let the scalars of nominal significance levels be 0.05, choose the lagged difference in {1, …, 3} by AIC, and assume that there are intercepts and linear trends in the cointegrating relations and there are quadratic trends in the data. The values equal to 1 indicate cointegration, and 0 indicate not.

In general, correlation analysis for non-stationary series probably gives spurious results, unless the series are cointegrated [22]. Hence the cointegrations in Table 2 guarantee the validity of the correlation analysis: the Spearman’s rank correlation coefficients [23] and the Pearson product-moment correlation coefficients [24] show that the development of algorithms are positively correlated with that of the mentioned research paradigms and transdisciplinary topics (Table 3).

**Table 3:** The correlation coefficients of certain time series pairs.

	model	experiment	simulation	data	system	network	control
experiment	0.95/1.00						
simulation	0.95/0.98	0.88/0.97					
data	0.95/1.00	0.98/1.00	0.88/0.97				
system	0.97/1.00	0.97/1.00	0.92/0.97	0.97/1.00			
network	0.96/0.98	0.89/0.97	0.94/0.99	0.88/0.97	0.93/0.97		
control	0.97/1.00	0.98/1.00	0.90/0.97	0.97/1.00	0.98/1.00	0.91/0.97	
algorithm	0.94/0.99	0.90/0.99	0.92/0.99	0.91/0.99	0.92/0.99	0.90/0.98	0.92/0.99

In each table cell, the first value is the Spearman’s rank correlation coefficient, and the second value is the Pearson product-moment correlation coefficient.

**Fig 5:**
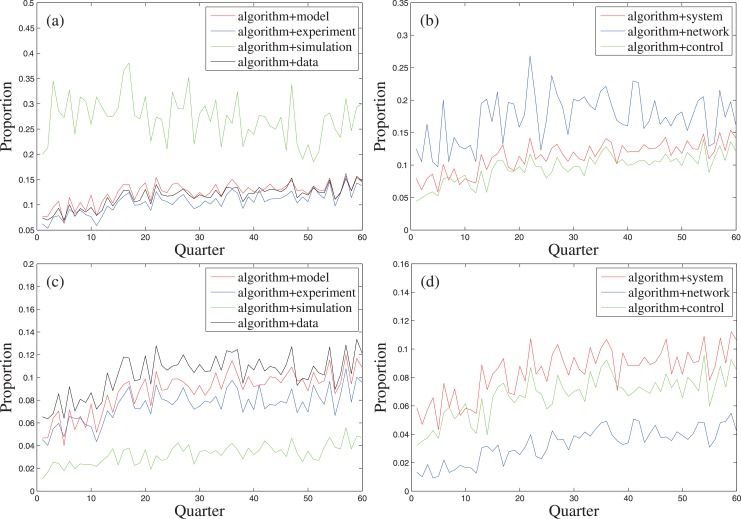
The quarterly proportions of the papers containing “algorithm” and a certain topic word amongst the papers containing that word (Panels (a,b)), and amongst all of the papers (Panels (c,d)).

The co-word occurrence analysis is also an efficient method to measure the relationship between topic words, which is based on the assumption that a paper containing two topic words means the topics expressed by the words are used or discussed by that paper simultaneously [19]. The proportions of the papers simultaneously containing “algorithm” and an aforementioned topic word amongst the papers containing that word, and amongst all of the papers are calculated respectively, annually and quarterly (Fig 5). The time series needed for the calculation are listed in S2 Table. The positive slopes of the linear fitting of the annual proportions (Table 4), except “algorithm” + “simulation” in “simulation”, show the increasing trends of algorithmization of the research paradigms and the methodologies given by the transdisciplinary topics. The reason for this exception is that the slope of the linear fitting of the annual proportion of the papers containing “algorithm” in all of the papers (0.0030) is lower than that of “simulation” (0.0064).

**Table 4:** The slopes of the linear fitting of certain time series.

model	experiment	simulation	data	system	network	control
0.0030	0.0037	-0.0028	0.0037	0.0040	0.0028	0.0038
0.0032	0.0021	0.0014	0.0024	0.0027	0.0022	0.0024

The time series are the annual proportion of papers containing “algorithm” and a certain topic word (the column heading) amongst papers containing that word (the first row), and amongst all of the papers (the second row).

Those cointegrations, positive correlations and increasing trends of algorithmization appear naturally and can be considered as some causes for the interdisciplinarity of applied mathematics. As simplifications of relevant aspects of research problems, models are generally described by mathematical concepts and language for systematic study [6]. Simulation, especially numerical simulation, has become a common method to algorithmically test how well the models are coherent to the experimental results. The widespread availability of computers and economic considerations make many of today’s sciences increasingly rely on simulation via mathematical models and algorithms. The scale of the data collected or generated from experiments and simulations can only be analyzed by algorithms [8, 9]. In fact, today’s science is becoming data-driven at a scale unimagined. Meanwhile, the theories of algorithms now guide researchers in mining the results from the collected data [25].

System science gives a unified methodology to research the complexity in epistemology by expressing the complex phenomena as complex systems, thus it is considered a transdisciplinary discipline [26]. A variety of abstract complex systems are studied as a field of mathematics. Ignoring the functionalities and characteristics of the original systems, systems can be investigated by abstracting them as networks. Researchers from different fields can investigate their respective problems under the unified network framework [12]. Algorithms play an important role in the analysis of the topological properties of the networks, such as distance and centrality finding algorithms, graph partitioning and clustering algorithms, and so on [27, 28].

Understanding of a system is reflected in our ability to control it. Control theory has a distinctly transdisciplinary mission to provide theories and approaches for comprehending complex phenomena [11]. The modern study of control uses various mathematical theories and approaches, such as neural networks, Bayesian probability, fuzzy logic, evolutionary computation, etc., which are all closely related to algorithms, e.g. genetic algorithms [29, 30].

The connections between applied mathematics and other disciplines are not only caused by algorithms, but also by some other mathematical topics. In fact, certain mathematical topics words, such as “equation”, “statistic” can be found in S1 Text. The quantitative analysis of the relationships between them and research paradigms or methodologies can be discussed as above, so is not addressed here.

## Conclusion

The interdisciplinarity of applied mathematics is quantitatively analyzed by using statistical and network methods on the corpus PNAS 1999–2013. A network is built based on the discipline information of the corpus, which gives a panoramic view of the relationships between disciplines. Some network indicators, e.g. betweenness centrality, quantitatively described the hub role of applied mathematics in interdisciplinary research. The statistical analysis on the corpus content found that a primary topic of applied mathematics, algorithms, cointegrates, correlates, and increasingly co-occurs with certain typical research paradigms and methodologies. Those findings can be considered as some of the underlying causes of the interdisciplinarity of applied mathematics.

## Supporting Information

S1 Table The quarterly number of papers in total (papers) and the quarterly number of papers containing a certain topic word in PNAS 1999–2003. (PDF)

S2 Table The quarterly number of the papers simultaneously containing “algorithm” and a certain topic word in PNAS 1999–2003. (PDF)

S1 Network The weighted discipline network data. (TXT)

S1 Text The wordlist of the corpus: PNAS 1999–2013. It includes the words appearing in the corpus and the frequencies of the occurrences of those words.
(TXT)
