# Supplementary material for: Quantitative Analysis of the Interdisciplinarity of Applied Mathematics
Source: PLoS One. 2015 Sep 9;10(9):e0137424. doi: 10.1371/journal.pone.0137424 (PMC4564225; doi:10.1371/journal.pone.0137424)
Supplement: S1 Table — (PDF) [file pone.0137424.s001.pdf]

The quarterly number of papers in total (papers) and the quarterly number of papers containing a certain topic word in PNAS 1999–2003.

papers	system	network	control	simulation	model	experiment	algorithm	data
749	554	80	543	40	456	546	52	663
597	452	57	425	47	361	448	39	545
747	543	86	510	55	505	543	61	659
654	499	57	477	56	430	497	56	600
671	495	72	476	44	429	493	44	615
685	515	75	484	55	428	496	66	627
610	469	76	442	50	383	468	47	557
668	507	70	463	51	420	481	60	582
770	588	109	540	59	482	558	61	700
600	443	80	437	54	385	444	55	548
735	560	92	510	51	485	544	57	671
640	484	76	455	58	408	470	55	566
721	550	113	517	80	501	522	79	654
882	677	139	605	120	589	636	84	800
607	447	102	428	48	416	439	67	561
831	613	127	571	82	555	600	104	763
632	449	83	451	63	434	466	83	577
794	568	112	583	65	554	585	82	730
692	514	103	476	63	467	499	76	626
739	572	120	512	86	508	553	92	674
839	643	146	623	71	585	639	86	777
931	706	138	692	106	636	690	127	848
777	588	94	559	78	539	573	90	717
705	534	130	490	95	508	527	81	640
882	644	155	638	102	617	671	106	822
800	611	143	550	117	555	577	99	715
833	651	144	608	100	609	631	107	760
904	710	174	673	105	655	684	102	836
930	720	184	693	100	695	726	102	864
870	682	169	627	107	651	669	102	809
807	627	151	590	98	589	593	88	734
936	721	190	706	117	681	719	101	867
1008	787	203	754	117	746	746	130	929
959	761	227	688	140	733	714	107	878
793	635	178	586	108	599	599	103	726
974	739	217	702	157	744	726	133	896
907	667	186	649	102	673	664	116	841
706	491	148	508	93	513	528	71	631
1160	867	233	773	176	870	810	139	1056
1089	800	231	732	146	796	744	124	950
1005	735	222	691	130	754	728	127	868
994	731	216	687	160	707	680	119	833
1112	799	243	735	172	793	764	136	934
1033	735	250	711	140	796	695	124	864
1123	813	261	808	150	826	787	133	914
1086	801	252	721	159	807	707	131	910
944	707	207	667	130	693	667	129	783
1043	750	231	722	160	789	696	115	853
1005	737	238	716	137	764	683	115	807
1021	713	220	676	164	742	669	107	839
1072	763	255	719	162	817	732	131	884
961	695	221	658	127	704	659	113	800
1008	740	246	698	142	765	682	115	832
1119	825	263	766	188	843	742	157	951
1037	738	248	700	160	801	707	104	840
1132	820	306	748	180	859	779	131	938
1058	758	237	715	190	808	702	146	894
997	735	282	682	150	739	683	113	803
1078	787	299	736	177	819	754	151	920
1141	857	299	800	180	853	793	156	942

The quarterly number of the papers containing a certain topic word, e.g. "model", is denoted by that word.
