# Supplementary material for: Quantitative Analysis of the Interdisciplinarity of Applied Mathematics
Source: PLoS One. 2015 Sep 9;10(9):e0137424. doi: 10.1371/journal.pone.0137424 (PMC4564225; doi:10.1371/journal.pone.0137424)
Supplement: S2 Table — (PDF) [file pone.0137424.s002.pdf]

The quarterly number of the papers simultaneously containing "algorithm" and a certain topic word in PNAS 1999–2003.

system	network	control	model	experiment	simulation	data
44	10	24	35	34	8	49
28	6	21	28	24	10	38
43	14	28	48	41	19	51
43	6	28	46	39	16	56
29	7	25	27	32	12	43
52	15	38	49	45	18	63
36	8	36	33	39	12	43
48	10	34	44	43	16	54
41	14	43	43	45	18	60
35	10	37	46	34	14	52
42	12	33	38	32	16	53
35	8	26	43	36	17	50
64	22	47	61	51	22	75
63	28	35	61	57	33	78
50	17	40	53	47	14	63
72	27	61	78	70	30	98
59	11	48	61	58	24	74
55	22	53	61	58	18	77
47	20	43	62	50	17	68
65	19	50	73	59	27	88
65	26	55	63	57	16	79
100	37	81	98	87	29	119
64	19	55	69	63	21	86
62	16	48	63	56	20	75
68	26	51	87	67	33	97
77	34	49	79	65	34	89
86	30	68	80	76	29	100
83	33	65	83	72	37	100
76	27	62	79	67	22	97
82	34	61	81	65	30	97
69	30	58	68	64	29	85
77	39	59	85	73	31	99
102	39	85	105	84	36	120
86	42	70	86	69	30	102
80	38	69	82	72	30	98
104	48	90	112	95	41	119
90	36	76	93	82	33	113
50	25	52	63	49	20	67
113	38	84	117	94	44	129
96	37	73	100	78	35	116
89	51	70	94	94	36	117
88	49	73	93	72	44	105
105	38	79	112	85	44	122
92	42	71	103	78	35	112
102	52	86	107	89	41	115
105	41	76	111	84	33	121
101	36	78	103	85	44	120
86	37	76	101	72	35	97
95	42	79	99	79	26	100
84	40	67	90	71	36	100
105	39	86	112	100	30	118
87	39	73	91	80	26	100
92	49	71	98	77	39	103
122	54	107	129	108	53	142
81	32	62	89	69	40	93
99	41	83	107	96	42	116
114	51	93	127	114	59	136
90	49	73	94	78	37	103
121	59	100	126	108	52	144
121	48	97	124	109	54	138

The quarterly number of the papers containing a certain topic word, e.g. "system", is denoted by that word.
